# ADAPT: a programme for the advanced detection of AI-enabled pathogenic threats

**DOI:** 10.3389/fbioe.2026.1819372

**Published:** 2026-06-09

**Authors:** Hanna Palya, Cassidy Nelson

**Affiliations:** 1 Institute for Global Pandemic Planning, University of Warwick, Coventry, United Kingdom; 2 Centre for Long-Term Resilience, London, United Kingdom

**Keywords:** Biodefense policy, biological AI models, dual-use research of concern (DURC), function-based screening, gene ontology (GO), protein language models, sequences of concern (SOC), synthetic nucleic acid screening

## Abstract

Advances in AI are expanding both the ceiling and the accessibility of biological engineering, creating threats that existing synthetic nucleic acid screening is not equipped to detect. The IARPA-funded Functional Genomic and Computational Assessment of Threats (FunGCAT) programme advanced screening by creating tools specialised for sequence screening and by progressing on the annotation of potential sequences of concern. However, 3 years after the conclusion of FunGCAT, critical gaps remain: (1) the field lacks an operationalisable definition of what makes a sequence a biosecurity concern, and (2) current tools cannot detect threats on the basis of function rather than sequence similarity. To close these gaps, we propose the Advanced Detection of AI-enabled Pathogenic Threats (ADAPT) programme in two phases as a successor to FunGCAT. ADAPT Phase I would develop a multi-attribute, function-based definition of sequences of concern and generate the benchmark datasets. Phase II would develop and validate screening tools capable of detecting known threats, AI-paraphrased functional homologues, and, where possible, AI-designed novel threats. Continuous governance workstreams would translate technical outputs into regulatory guidance and maintain secure infrastructure. ADAPT builds on FunGCAT’s legacy and the subsequent work of the synthetic nucleic acid screening community, while adapting to an era in which biological AI models can generate functional sequences bearing little resemblance to any previously characterised sequence.

## 1 Introduction

AI is transforming biological engineering in two ways. Large language models (LLMs) are democratising access to biological knowledge, while specialised biological AI models (BAIMs) push the frontier of protein and genome design. Increasingly, LLMs and BAIMs are being combined into tools that do both (Justen, 2025; Anthropic, 2025; Madani et al., 2023; Hayes et al., 2025; De Almeida et al., 2025; Huang et al., 2025). This trajectory of capabilities calls for corresponding advances in synthetic nucleic acid screening, a critical boundary between digital biological design and physical biological material.

The most significant prior investment in synthetic nucleic acid screening was the Intelligence Advanced Research Projects Activity (IARPA) programme “Functional Genomic and Computational Assessment of Threats” (FunGCAT) (Intelligence Advanced Research Projects Activity, 2022). FunGCAT produced three screening tools, new ontologies for pathogenic functions, and annotations for many previously unannotated viral genes (Balaji et al., 2022; Beal et al., 2021; Battelle, 2018; Godbold et al., 2022; Ng et al., 2022; Gemler et al., 2022). These results reduced the minimum detectable sequence length from gene-length to oligo-length, and took initial steps from purely taxonomic matching towards function-based characterisation of threats.

FunGCAT aimed to detect uncharacterised pathogenic functions, whether arising from naturally evolved sequences or from engineered modifications of genes with known wild-type sequence and function. The threats it aimed to defend against were ‘bio-error’ and ‘bio-terror’: unconsidered dual-use research and deliberate attacks (IARPA, 2016a; IARPA, 2016b). Since 2016, these threats have expanded substantially. We therefore propose the Advanced Detection of AI-enabled Pathogenic Threats (ADAPT) programme as a successor to FunGCAT, designed for an era in which AI can generate functional biological sequences with little or no homology to known threats.

## 2 Advances in synthetic nucleic acid screening

In a report in April 2025, the National Academy of Sciences claimed that “current AI-enabled biological tools are not capable of the *de novo* design of a self-replicating organism such as a virus” (NASEM, 2025). However, in September 2025, the first viable, fully AI-generated genomes were published based on the Φ X174 bacteriophage (King et al., 2025). While these genomes retain high similarity to wild-type sequences and are detectable by taxonomy-based screening, they show a trajectory of capability that current screening frameworks were not designed for. The developments in LLMs and BAIMs are prompting revisions in synthetic nucleic acid screening policy to safeguard the boundary between the digital and physical (National Science and Technology Council, 2024; The White House, 2025).

Nucleic acid synthesis policy first emerged as voluntary industry standards by the International Gene Synthesis Consortium (IGSC) in 2009 (IGSC, 2009). Since then, the IGSC have updated their screening protocol twice, responding to advancements in benchtop DNA synthesis devices, sequence screening technology, and AI (IGSC, 2017; 2024). Members of the IGSC have also supported the development of national and international policies on nucleic acid synthesis, the first of which was the 2010 US HHS Screening Framework (HHS, 2010). We compare this with the three most recent policies from the US, UK and the IGSC in Appendix Table 1 (HHS, 2010; 2023; IGSC, 2024; DSIT, 2024).

**TABLE 1:** Proposed structure of the ADAPT programme, showing workstreams organised into two phases and a continuous governance stream.

Workstreams	Activities
Phase I. Definition and test set development (Years 1–2)	
I.1	Develop an ontology that defines sequences of concern as clusters of weighted attributes, including but not limited to molecular function, necessity for pathogenicity, host range, evidence strength, and weaponisation potential
I.2	Populate I.1 ontology through annotation from published studies, PLM-based functional prediction, and high-throughput functional assays
I.3	Adapt and extend the NIST nucleic acid screening test set to reflect the definition developed by Workstream I.1. The test set should include: (a) known threats with established ground truth, (b) AI-designed and paraphrased sequences, and (c) benign sequences
Phase II. Screening tool development and evaluation (Years 2–5)	
II.1	Develop screening tools, including adaptation of existing tools and novel approaches using BAIMs
II.2	Synthesise and functionally characterise edge-case sequences like novel AI-generated sequences identified by II.3 red-teaming as potential evasions and sequences where Phase II tools disagree and no ground truth exists
II.3	Evaluate all screening tools against the Phase I test set and conduct adversarial testing with AI-designed sequences. The results should feed back to II.1 and II.2
Governance workstreams (Years 1–5)	
G.1	Translate technical outputs into regulatory guidance for relevant bodies (in the US: HHS, OSTP; in the UK: DSIT; internationally: IGSC, WHO), mapping the ADAPT definition to existing frameworks and identifying gaps requiring new regulation
G.2	Design and deploy secure infrastructure for the ontology, test sets, and screening tools

To address the persistent challenge of defining and screening for sequences of concern, the FunGCAT programme ran from 2017 to 2022 and resulted in new ontologies for pathogenic sequences, the annotation of eukaryotic toxins, fungal, viral and bacterial SoCs and the development of three screening tools: FAST-NA, SeqScreen and ThreatSEQ, each improving screening capabilities (Balaji et al., 2022; Beal et al., 2021; Battelle, 2018; Godbold et al., 2022; Ng et al., 2022; Gemler et al., 2022). This allowed guidance to move from screening from BLAST-based best-match screening of 200 bp sequences to taxonomic and limited functional screening of 50 bp sequences (IGSC, 2017; HHS, 2023). We provide an overview of the tools in the Supplementary Material.

All three groups continued advancing their tools after FunGCAT concluded, addressing new challenges in synthetic nucleic acid screening (Wittmann et al., 2025). Further, after the conclusion of FunGCAT, three new screening tools were developed: the Common Mechanism, SecureDNA and Aclid (Wheeler et al., 2024; Baum et al., 2026; Aclid, 2026). The programme thus directly led to the development of screening tools, and had broader effects in building a networked community of experts, who continue to work towards standardised screening (Laird et al., 2025).

## 3 Remaining gaps in synthetic nucleic acid screening

Despite the significant progress made by the FunGCAT programme and subsequent efforts, critical gaps in synthetic nucleic acid screening remain (Gillum and Moritz, 2025; Rose et al., 2024; Gemler et al., 2024; Mackelprang et al., 2025). These gaps present security risks and leave ambiguity for policymakers and commercial DNA providers, leading to uneven detection capabilities across providers. We categorise these as definitional gaps (what properties to screen for) and methodological gaps (how to screen for them).

### 3.1 Defining sequences of concern - what to screen for?

A persistent challenge for screening is the lack of an unambiguous and operationalisable definition of what constitutes a sequence of concern (SoC) (Gillum and Moritz, 2025; Godbold et al., 2025b; Rose et al., 2024). Without it, governments cannot create enforceable frameworks that move beyond taxonomic screening, causing regulatory paralysis. It also creates a difficult operating environment for synthetic nucleic acid providers, who are often left to interpret vague guidance (Gemler et al., 2024; Sharkey et al., 2024). Without a clear definition, screening pipelines suffer from false positives, which lead to expensive follow-up and hinder wider adoption, and false negatives, which pose a direct biosecurity risk.

An operationalisable definition of SoCs that remains stable in the face of novel threats requires multiple dimensions: the nature of the encoded function and its relationship to pathogenicity, the taxonomic context, and the potential for weaponisation. A central challenge is determining which functions are concerning, as it is unclear where to draw the line on what counts as contributing to harm (Elworth et al., 2020). Existing efforts to address this include the introduction of new GO-terms for describing the harmful effects of proteins on host biological processes, and comparative genomics for identifying signatures of pathogenicity. However, these cover only a fraction of known pathogenic functions and remain limited by sparse experimental evidence on gene essentiality and sufficiency for pathogenicity (Godbold et al., 2025a; Beal et al., 2021; Torres et al., 2024; Elworth et al., 2020).

Beyond biological process functions, a complete SoC definition must also account for other threat-relevant attributes of sequences, including weaponisation potential and taxonomic context, both of which remain essential for mapping onto existing regulatory frameworks (Godbold et al., 2023; Mackelprang et al., 2025; Federal Select Agent Program, 2025; The Australia Group, 2023; UK Parliament, 2001; Godbold et al., 2025b; Siddiqui et al., 2025). A multi-dimensional definition becomes increasingly important as BAIMs gain the ability to produce sequences with the functions of known SoCs without conserving sequence identity (Kobokovich et al., 2019; Elworth et al., 2020; Gillum and Moritz, 2025; Mackelprang et al., 2025), making comparison on any single dimension insufficient for reliable screening.

### 3.2 Detecting the whole spectrum of threats - how to screen?

Once a definition of SoCs is developed, tools capable of evaluating sequences against this definition will need to be developed. Multiple tools will likely need to be arranged in a cascade to form a method capable of screening a wide spectrum of sequences (Wheeler et al., 2024; Wittmann et al., 2025). Relating AI-generated sequences to the SoC definition will require different computational approaches than for exact matches or synthetic homologs of known sequences.

Known Threats: Screening for the exact DNA sequences of known pathogens (e.g., smallpox) is a largely solved problem for viruses, with the exception of very short sequences of fewer than 30 basepairs (Laird et al., 2025). Short sequences could be used to obfuscate orders by splitting them into small fragments, each of which is too short to be an exclusive match to known pathogens but can be joined to get a sequence of concern (DiEuliis et al., 2017; Biodefense in the age of synthetic biology, 2018). Bacterial and eukaryotic sequences of concern are harder to identify, but this is mostly due to the lack of annotation (Godbold et al., 2025b; Elworth et al., 2020).

“Paraphrased” Threats: This category includes sequences with different codons that translate to the same amino acid sequences, or AI-redesigned proteins that are functionally identical to known proteins but have different amino acid sequences. The first can be addressed by translating the nucleic acid sequence into amino acids and screening those resulting proteins (HHS, 2010). Wittmann et al. (2025) demonstrated the second vulnerability by using AI to paraphrase known toxins in ways that evaded screening, resulting in synthetic homologs.

Novel Threats^1^: This category includes AI-designed proteins that might have entirely novel ways of affecting biological processes such that they cannot be traced back to known threats, like paraphrased threats can. This is something ADAPT cannot directly defend against but will have to anticipate (Baker and Church, 2024; Hunter, 2024). Novel functions will have to be added to a collection of functions of concern, with limited access. ADAPT’s defence against genuinely novel threats is therefore indirect: the multi-attribute ontology and governance workstreams are designed to absorb new functional categories as they are characterised in the literature or by red-teaming, but detection of mechanisms with no representation in existing functional space remains a research frontier that ADAPT anticipates rather than solves.

## 4 Filling the gaps in synthetic nucleic acid screening

ADAPT would address the questions of (a) what to screen for and (b) how to screen in turn. FunGCAT’s approach of combining functional characterisation of threats with building tools to detect them should remain, while the methods used should change, given the development of large biological models and function prediction tools (Hayes et al., 2025; Nguyen et al., 2024; Sanderson et al., 2023; Kulmanov and Hoehndorf, 2020; Yu et al., 2023).

### 4.1 Creating a multi-attribute ontology for sequences of concern

The first task of ADAPT would be to define what sequence properties constitute a SoC, enabling synthetic nucleic acid screening to move from static lists to a living ontology of pathogenic functions. The biological processes GO terms and the annotations by Godbold et al. are a helpful starting point for determining what functions to screen for (Godbold et al., 2025a). However, current annotations cover only a fraction of known pathogenic functions (Balaji et al., 2022). The scale of the remaining task, spanning thousands of viral, bacterial, and eukaryotic genes, far exceeds what manual curation alone can achieve. Computational approaches are therefore essential to populate the ontology at the breadth and speed required. However, these pathogenic functions occupy rare, peripheral nodes in the GO graph. CAFA community benchmarks show that protein function predictors perform substantially worse on rare GO terms than on common ones. Therefore, this annotation work should be done through a combination of wet-lab validation to generate new ground truth, large-scale annotation with predictions from large biological models, and systematic scraping of published studies for annotation and re-annotation.

Protein language models capture functional signals in sequence statistics and can aid annotation work. PLM-based embeddings have increased annotated viral protein fractions by 29% (Flamholz et al., 2024), served as input features for hierarchical multilabel classifiers that assign interrelated GO terms (Shao et al., 2024), and enabled multimodal models to generate natural-language functional descriptions directly from sequence (Flamholz et al., 2024; Shao et al., 2024; Xu et al., 2023; Li et al., 2025). These approaches can delineate functions of concern at multiple levels of biological organisation, with the ultimate goal of providing a definition of SoCs that is operationalisable.

Such a definition will likely take the form of a weighted, hierarchical cluster of attributes, where concern is assessed by converging evidence rather than a single binary flag. Weighting could reflect the strength of experimental evidence linking a function to pathogenicity, the necessity or sufficiency of the sequence for that function, and the potential for weaponising a pathogen with that function through enhancing transmissibility, immune evasion, or resistance to medical countermeasures. This multi-attribute definition would allow screening to distinguish, for example, between a housekeeping gene shared across pathogenic and non-pathogenic organisms and a virulence factor essential for host harm. The virulence factor would score highly on necessity for pathogenicity and strength of experimental evidence, while the housekeeping gene would score low on both despite taxonomic overlap with a pathogen. Assessing concern through converging evidence across weighted attributes rather than a single flag would reduce false positives from shared housekeeping functions and false negatives from AI-redesigned virulence factors that evade sequence-similarity screening. This cluster could pose an information hazard, so we recommend that ADAPT include a secure, managed-access database to host it.

### 4.2 Developing new sequence screening tools

BAIMs used to curate functions of concern could also be fine-tuned to screen sequences. Protein language models embed sequences into high-dimensional representations where function clusters can be observed even when sequence similarity is low (Kaminski et al., 2023). Screening could measure proximity between a candidate and known functional classes, similar to FAST-NA’s approach but based on functional annotations instead of the pathogenicity of the biological agent. Models trained across many biological functions have been shown to generalise to new ones after fine-tuning on tens of examples in the case of toxins (Zhou et al., 2024). If this few-shot capability works for other SoC categories, screening for newly identified functions could begin before large annotated training sets are available.

Deployable tools should take the form of cascading pipelines that combine screening based on multiple sequence attributes. Fast methods would screen everything; expensive tools like pLM-based structural analysis would apply only to sequences unresolved in earlier steps. This architecture would keep false positivity rates manageable, as each stage would refine the previous one. Current screening tools could form the earlier parts of these pipelines. Wittmann et al. showed that they could be altered to recognise functional homologs from their mostly toxin test set. After changing its algorithm, the tool that performed worst initially went from flagging 31% to flagging 99% of homologs with high *in silico* metrics. Two other tools, which flagged more than 80% of these homologs, achieved 97%–98% sensitivity after modification (Wittmann et al., 2025). However, Wittmann et al. caution that this may not remain the case as BAIMs improve. Still, current screening tools could serve as starting points for scaling more advanced functional prediction.

The role of ADAPT will be to facilitate the development and evaluation of function-based screening tools at scale. We outline example projects in Supplementary Table S2, organised into two tracks: (1) defining SoCs through sequence- and structure-based motif discovery, representation learning on protein language models, and wet-lab validation of AI-redesigned proteins; and (2) detecting these features at scale using approaches including few-shot classifiers on protein language model embeddings, graph neural networks over the Gene Ontology, and hybrid sequence-structure methods. As the screening tools themselves are dual-use, they must be hosted in environments that keep the threat-detection logic secure. They will also have to be privacy-preserving, as the sequences submitted for synthesis are often proprietary. For computationally expensive components like protein language models fine-tuned to predict threat signatures, this may require either secure enclaves at provider sites or architectures in which providers submit sequence embeddings rather than raw sequences to centrally held classifiers. ADAPT could produce an encoder trained jointly with the screening classifier and distribute it to providers. An encoder trained end-to-end with the screening classifier would learn representations tailored to the classification task. However, compressing sequences into fixed embeddings introduces an information bottleneck, and some accuracy loss relative to full-sequence screening is expected. Characterising this trade-off would be a necessary part of Phase II evaluation.

## 5 Proposed structure of ADAPT

Given the expanded biosecurity threats from AI, the potential for progress in function-based screening, and the need for harmonised efforts, we recommend a defence- or intelligence-funded programme for the Advanced Detection of AI-enabled Pathogenic Threats (ADAPT). This 5-year programme would aim to create a multi-attribute definition of SoCs, generate wet lab data to support annotations, build tools that screen based on this definition, and set up a system to continuously advance screening systems as novel threats emerge.

**FIGURE 1:**
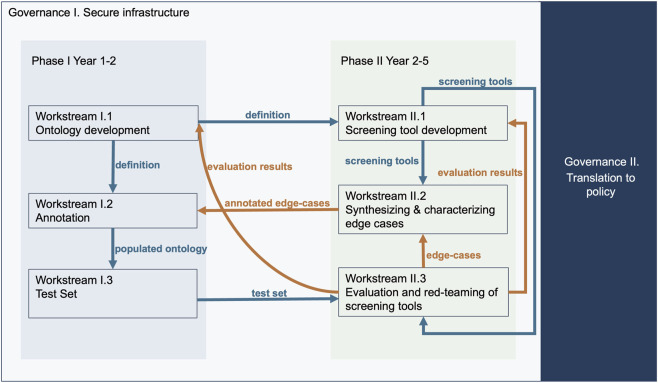
Proposed structure of ADAPT, with workstreams feeding into each other.

ADAPT would consist of two phases and a continuous governance stream. Phase I would focus on creating a multi-attribute, function-based definition of SoCs. Phase II would focus on developing tools based on this definition (Table 1; Figure 1).

We estimate that the budget needed for ADAPT sits between a large ARIA programme and a DARPA programme ($65-$80M; £50-£60M) with performers including computational biologists, virologists, immunologists, software engineers and policy researchers (ARIA, 2024; Defense advanced research projects agency, 2025; Global Biodefense, 2021). The phased structure allows for course correction, while the continuous governance workstreams ensure that ADAPT establishes infrastructure for ongoing adaptation to an evolving threat landscape. A breakdown of the programme and its budget is available in the Supplementary Material.

The case for launching ADAPT now rather than deferring is grounded in the pace of AI-bio capability development. The interval between the publication of the first viable fully AI-generated genomes and the anticipated deployment of next-generation biological design tools is narrowing (King et al., 2025). New generations of BAIMs will produce sequences that are harder to relate to known threats through sequence similarity alone (Wittmann et al., 2025; Baker and Church, 2024). Establishing the definitional and computational infrastructure for function-based screening before these capabilities mature widely is easier and less costly than attempting to retrofit defences after a screening failure.

The design of ADAPT raises several security considerations. We propose it as a defence-funded programme to provide secure environments for red-teaming. Further, the ontology developed by ADAPT has dual-use risks by cataloguing pathogenic functions and evasion strategies. At the same time, because screening is only as strong as the weakest compliant jurisdiction, uptake must extend beyond the regulatory environments for which ADAPT is primarily scoped (the US and UK). Two near-term routes exist: the IGSC as an industry-led voluntary consortium, and ISO 20688-2:2024, the international standard for synthesised nucleic acids that already includes biosecurity screening provisions and is referenced by UK DSIT guidance (International Organization for Standardization, 2024; DSIT, 2024). The Governance II workstream should engage with both—informing updates to the IGSC Harmonized Screening Protocol and feeding ADAPT’s ontology and tools into revisions of ISO 20688-2 — so that outputs are accessible to providers under both statutory and voluntary regimes, including in jurisdictions with substantial synthesis capacity such as China and India. To balance these requirements, we propose a tiered access framework, restricting sensitive data to vetted providers and regulators under the oversight of a standing biosecurity review panel, while keeping screening tools broadly accessible.

ADAPT is scoped for sequence screening at commercial synthetic nucleic acid providers. Benchtop DNA synthesisers currently enable oligo-length sequence production outside of these providers, and might enable gene-length production in the future. The function-based screening tools developed by ADAPT are not inherently limited to this setting. If the policy environment moves beyond customer-only screening for benchtop devices—for example, through “phone-home” architectures in which instruments query a remote screening service before initiating synthesis—the products of ADAPT could serve as the back-end.

The programme also faces practical limitations. Cost estimates are uncertain, the 5-year timeline may need revision if AI advances faster than anticipated, and function-based screening is difficult to validate when ground truth is sparse. Wet-lab characterisation of edge cases (Workstream II.2) provides independent ground truth, but the space of possible novel functions will, for the foreseeable future, exceed what can be experimentally validated. ADAPT therefore treats validation as iterative, with the governance workstream updating the ontology and benchmarks as new evidence emerges. Despite these challenges, the programme’s first output, a well-defined SoC ontology, has standalone value, and even adoption within a single jurisdiction would raise the floor for screening quality among compliant providers.

## 6 Conclusion

For synthetic nucleic acid screening to be a robust defence against AI-enabled biothreats, two gaps must be closed. First, the field needs an operationalisable definition of what makes a sequence concerning. Without this, regulators cannot enforce minimum screening standards and providers cannot build consistent screening pipelines. Second, new tools are needed that can relate non-homologous sequences to this definition, from sequences with high homology through sequences paraphrased with BAIMs to sequences with entirely novel functions. ADAPT addresses these gaps in sequence: Phase I establishes the definition and builds the benchmark; Phase II develops and validates the tools. A continuous governance workstream ensures harmonisation between the two phases and with evolving regulation.

ADAPT builds on FunGCAT’s legacy while adapting to the current reality and anticipated future of AI-enabled biological design. A coordinated, well-resourced effort can establish the infrastructure needed to maintain effective oversight as AI-bio integrations develop.

## Data Availability

The original contributions presented in the study are included in the article/Supplementary Material, further inquiries can be directed to the corresponding author.

## Footnotes

1 The distinction between “paraphrased” and “novel” threats is best understood as a spectrum across levels of biological organisation rather than a binary. A synthetic protein with a redesigned amino acid sequence that retains the same enzymatic mechanism as a known virulence factor is paraphrased at the functional level. One that achieves the same pathological outcome through a different molecular mechanism, for example, through disrupting host immune signalling through a novel binding interaction rather than a known one, occupies an intermediate position. One that produces a harmful phenotype through a mechanism not represented in any existing functional category is genuinely novel.
