# Supplementary material for: ADAPT: a programme for the advanced detection of AI-enabled pathogenic threats
Source: Front Bioeng Biotechnol. 2026 Jun 9;14:1819372. doi: 10.3389/fbioe.2026.1819372 (PMC13286858; doi:10.3389/fbioe.2026.1819372)
Supplement: Supplementary file 1 [file DataSheet1.pdf]

Supplementary Material to ADAPT

1 SCREENING GUIDANCE

In addition to voluntary guidance, there have been attempts to mandate synthetic nucleic acid screening in several U.S. states, most of which were not passed (California State Legislature, 2021; Maryland State Legislature, 2021). AB1963 passed in California, requiring California State University and requesting the University of California to develop guidance on purchasing gene synthesis products and equipment from providers who prevent the misuse of these products (California State Legislature, 2022).

On the federal level, the first attempt at regulation was part of President Biden's Executive Order (EO) on AI in October 2023 (The White House, 2023). The order directed the White House OSTP to create the Framework for Nucleic Acid Synthesis Screening. The Framework was released in April 2024, and outlined the requirements providers needed to meet to be able to serve federally funded customers, based on the 2023 HHS Guidance (National Science and Technology Council, 2024). It also established a requirement to screen for functional SoCs from 13 October 2026. It came into effect on April 26, 2025. President Trump's EO on Improving the Safety and Security of Biological Research ordered that a revised framework replace the Biden-era one (The White House, 2025). However, as no revised framework has been published at the time of writing, most federal funding agencies continue to require compliance with the original framework in their grant agreements.

President Trump's EO maintained the principle of leveraging federal funding requirements for synthetic nucleic acid screening. It ordered the revision of the Framework with stronger, top-down oversight instead of self-reporting and called for a strategy to expand screening requirements to non-federally funded research (The White House, 2025). The revised Framework has not been released at the time of writing.

Table S1. Comparison of national and international guidance frameworks for synthetic nucleic acid screening.

	2010 U.S. HHS Screening Framework	2023 U.S. HHS Screening Framework	2024 UK Screening Guidance	IGSC Harmonised Screening Protocol v3.0 (2024)
**Scope**	Providers of synthetic dsDNA.	Providers, users, vendors, and manufacturers of benchtop synthesisers.	All UK individuals and organisations involved with synthetic nucleic acids and benchtop equipment.	IGSC Provider and Manufacturer members and their customers/users.
**Sequences of Concern (SoCs)**	Agents on U.S. Select Agent (BSAT) and Commerce Control (CCL) lists.	All sequences contributing to pathogenicity or toxicity (from regulated and unregulated agents).	Sequences contributing to pathogenicity, virulence, or toxicity, based on UK regulations (e.g., ATCSA Schedule 5).	Sequences from a Restricted Pathogen Database (RPD) including U.S., Australia Group, EU, and UK lists.
**Nucleic Acid Type**	Double-stranded DNA (dsDNA).	Single- and double-stranded DNA and RNA.	All synthetic nucleic acids (ss/ds DNA, RNA) of any length.	Single- or double-stranded DNA or RNA.
**Sequence Screening Window**	200 base pairs (bp).	50 nucleotides (nt) within 3 years; $<$50 nt for potential assembly.	Minimum of 50 nt; shorter sequences encouraged if assemblable into a SoC.	Currently $\geq$200 bp; transition to 50 bp threshold by Oct 2026.
**Customer Screening & Validation**	Recommended for all orders. Verify identity and screen against denied party lists. Follow-up on 'red flags'.	Required for orders with SoCs and for benchtop synthesiser purchases. Verify legitimacy via affiliation, publication history, etc.	Required for all orders. Verify identity via name, address, purpose, and another identifier (e.g., ORCID).	Required for all orders. Screen against denied party lists. Additional verification for regulated pathogen sequences.
**Record Keeping & Cybersecurity**	Retain records for 8 years.	Retain records for 3 years. Includes cybersecurity for SoC databases and benchtop devices.	Retain user and SoC order info for 3 years. Includes cybersecurity for customer data and screening process.	Retain product, delivery, and screening records for 8 years.

2 SCREENING TOOLS

2.1 SeqScreen

The only publicly available and most thoroughly documented sequence screening tool resulting from FunGCAT is SeqScreen (Balaji et al., 2022). SeqScreen was developed through a collaboration between Rice University and Signature Science. After categorising sequences of pathogenic microbes according to their functions to aid annotation, the SeqScreen team curated a training dataset of functions of sequences of concern (FunSoCs) (Godbold et al., 2022). This dataset of annotated sequences was then used to train an ensemble machine learning classifier that maps UniProt IDs to FunSoCs, which became the functional annotation step in SeqScreen's screening pipeline. The screening pipeline consists of several different workflows with varying levels of sensitivity and computational power requirements. The user can select from optional tools to best fit their screening requirements.

1. **Initialisation:** Checking for syntactic errors in the FASTA file and suspiciously long sequences, and translating the nucleotide sequences into amino acid sequences for the next steps.
2. **SeqMapper** (only sensitive mode): Aligning sequences to Biological Select Agents and Toxins (BSAT) sequences with Bowtie 2 and RAPSearch.
3. **Protein and taxonomic identification:** Using alignment-based tools to identify and predict the protein of origin (DIAMOND) and classify the taxonomy of the sequences (BLASTX, BLASTN).
4. **Functional annotation:** Matching the sequences to FunSoCs, using predicted UniProt IDs, DIAMOND bit scores, and a database mapping UniProt IDs to FunSoCs.
5. **Output:** An HTML report containing general sequence information, assigned FunSoCs, and gene of origin (if found).

According to their case studies, SeqScreen is suitable for screening for both known and novel pathogens. The software is available for biohazard detection from environmental surveillance through a collaboration with Oxford Nanopore. While SeqScreen is open-source software, Signature Science also developed an add-on to SeqScreen, named S2FAST, which is available for use by the U.S. Government and other government or international agencies approved by IARPA. It processes the SeqScreen outputs into customised assessments to determine the level of concern for each sequence in accordance with the specific screening policy of the user.

2.2 ThreatSEQ

ThreatSEQ (now UltraSEQ), developed by Battelle, is a proprietary screening tool underpinned by a curated gene-level database of sequences of concern spanning bacterial, viral, and eukaryotic pathogens, categorised by function. The tool aligns query sequences against this database and NCBI reference genomes, then classifies hits into threat tiers based on similarity to select agents and known SoCs (Battelle, 2018; Gemler et al., 2023). Twist Bioscience reportedly adopted ThreatSEQ in 2019 (Twist Bioscience, 2019).

2.3 FAST-NA

FAST-NA, a proprietary tool, was developed by RTX-BBN, adapting their Framework for Autogenerated Signature Technology (FAST), originally developed for malware detection in network traffic (Wyschogrod et al., 2022). FAST-NA generates diagnostic signatures by comparing k-mer sequences from controlled pathogenic organisms (target material) against closely related but non-controlled taxa (contrast material). For example, SARS and MERS sequences serve as target material, while benign human coronaviruses

229E and NL63 provide contrasting sequences. The system uses Bloom filters to store contrast signatures and systematically remove any target signatures that match contrast material, leaving only signatures diagnostic of biological threats.

When FAST-NA was tested on NCBI sequences, it demonstrated strong performance on viruses: no false negatives, a 0.039% probability of a sequence being assigned to multiple pathogens, and a 0.55% false positive rate. Screening for bacterial pathogens proved more challenging due to horizontal gene transfer, but false positive rates remained below 2%. The system also operates orders of magnitude faster than BLAST (more than 10 kilobases per second) with substantially lower computational requirements. FAST-NA has been adopted by Integrated DNA Technologies (IDT) and demonstrated similar or improved performance on live customer synthesis orders, validating its effectiveness outside of NCBI sequences.

3 POTENTIAL PROJECTS FOR DEFINING SOCS AND DEVELOPING SCREENING TOOLS

Table S2. Potential technical projects for improving synthetic nucleic acid screening.

Subtask	Type	Description	Key Output
**What to screen for?**			
FunSoC definitions	Comp.	Map concerning functions to GO terms	Unified functional ontology
Comparative motif analysis	Comp.	Align many proteins with same GO term; find conserved sequence regions across diverse proteins	Database of conserved functional signatures
Structure-based motif discovery	Comp.	AlphaFold 3/ESM-2 all proteins with same function; identify regions that fold into similar 3D geometry even when sequences differ	Structurally conserved motifs (5–25 aa)
Conservation analysis	Comp.	Use coevolution signals to find residues that change together; identify critical contacts	Context requirements for motifs
Motif validation	Comp.	Test if identified motifs actually predict function on held-out proteins	Validated motif–function associations
Motif validation	Lab	Synthesise conserved motifs as peptides; test activity alone and in different scaffolds	Proof that motifs confer function
Mutational scanning	Lab	Systematically mutate safe proxy proteins; measure which mutations preserve function	Sequence-to-function map
Protein validation	Lab	Test AI-redesigned non-pathogenic proteins; verify predictions match experimental activity	Ground truth for computational models
Sparse autoencoders	Comp.	Train on ESM-2 embeddings to find interpretable latent features	Novel motifs
Build test sets	Comp.	Curate known threats, AI-redesigned, fragments, hard negatives	Test set
**How to screen?**			
PLM embeddings + few-shot learning	Comp.	Use ESM-2 to embed sequences; train classifiers that generalise from 10–50 examples per function	Fast function predictor
Graph neural networks	Comp.	Model GO hierarchy as graph (PANDA2-style); propagate predictions through functional relationships	Hierarchical function prediction
CNN-based prediction	Comp.	ProteInfer-style direct GO term prediction from sequence	CNN prediction method
Motif scanning	Comp.	Database of comparative motifs + context checking with PLMs	Interpretable motif screening
Hybrid structure + sequence	Comp.	Combine PLM embeddings with AlphaFold for flagged sequences	Analysis of uncertain cases

4 ADAPT STRUCTURE AND BUDGET

We estimate the total cost of ADAPT at $65-78M (£50–62M), distributed across two phases and continuous governance. This is comparable in scale to DARPA's Nucleic Acids On-Demand Worldwide (NOW) programme and sits at the upper end of current ARIA programme budgets (£50-57M) (ARIA, 2024; dar, 2025; DNA Script, 2021). Phase I costs are driven by high-throughput functional assays for ontology population; Phase II costs are driven by BSL-3 wet-lab validation of edge-case sequences. All costs are in 2025 USD. Personnel costs assume a blended fully loaded rate of ˜$300K/yr (see assumptions below).

4.1 Phase I: Definition (Years 1–2, $15–19M)

Central coordination: A government or Federally Funded Research and Development Center (FFRDC)-style entity responsible for convening stakeholders, maintaining the evolving ontology, and hosting the secure reference database.

4.1.1 Workstream I.1 — Ontology for SoC definition

Activity: Defining 'sequence of concern' as a cluster of weighted attributes. Attributes include: function at multiple biological levels (molecular function, biological process, host effect), necessity and/or sufficiency for pathogenicity, host species and transmission potential, evidence strength (experimental vs. inferred), and weaponisation potential (enhancement of transmissibility, host range, or countermeasure resistance). The definition must be harmonisable with export controls and DURC guidance.

Performers: NIST, SBRC, computational biologists, immunologists, DNA providers, tool developers, and policy specialists familiar with existing regulatory frameworks.

Outputs: A tiered classification scheme and explicit decision rules for edge cases. The infrastructure should support rapid integration of new threat classes as they are identified.

Item	Cost	Reference
Personnel: 4 FTEs × 2 yr × $300K	$2.4M	2 computational biologists, 1 virologist, 1 ontology specialist. Blended FTE cost per assumptions (National Institutes of Health, 2024; ZipRecruiter, 2024; National Institute of Allergy and Infectious Diseases, 2024; Congressional Research Service, 2024; Association of Public and Land-grant Universities, n.d.)
Expert workshops, advisory panel, travel	$0.3M	3–4 workshops over 2 years; comparable to NAS/NASEM workshop costs ($50–100K per convening)
**I.1 Subtotal**	**$2.7M**	

4.1.2 Workstream I.2 — Annotation at scale

Activity: Populating the ontology with ground-truth labels through three complementary approaches: (a) annotation from published studies, (b) PLM-based functional prediction, and (c) high-throughput functional assays.

Performers: Computational biologists, immunologists, and wet-lab researchers.

Outputs: An expanded set of annotated sequences feeding the definition (I.1), the test set (I.3), and training data for Phase II tools.

Item	Cost	Reference
Personnel: 6 FTEs × 2 yr × $300K	$3.6M	2 bioinformaticians/annotators, 2 ML engineers (PLM work), 1 assay biologist, 1 coordinator (National Institutes of Health, 2024; ZipRecruiter, 2024; National Institute of Allergy and Infectious Diseases, 2024; Congressional Research Service, 2024; Association of Public and Land-grant Universities, n.d.)
GPU compute for PLM fine-tuning & inference	$0.3–0.5M	Fine-tuning ESM-2, ProtTrans, etc. H100 GPUs at $2–8/hr (GMI Cloud, 2025; RunPod, 2025); a single fine-tuning campaign on a 3B-parameter model costs ~$10–50K in compute; budget assumes dozens of runs plus batch inference
High-throughput functional assays	$4–7M	Dominant cost. Pooled CRISPR screens require large cell libraries and NGS readout (Target Discovery Institute, University of Oxford, n.d.; Bock et al., 2022). Gene synthesis at ~$0.09–0.20/bp (Bio Basic, n.d.; Carlson, 2025; Twist Bioscience, 2025); library of hundreds to thousands of constructs at 1–5 kb = $0.5–2M in synthesis. Cell-based assays, reagents, sequencing, and CRO fees add $2–5M
Literature annotation tooling	$0.3M	Software licences, NLP pipeline development, database infrastructure
**I.2 Subtotal**	**$8–11M**	

4.1.3 Workstream I.3 — Test set

Activity: Adapting and extending the NIST nucleic acid screening test set to reflect the definition developed by Workstream I.1. The test set should include: (a) known threats with established ground

truth, (b) 'paraphrased' sequences (codon-optimised variants, AI-redesigned functional homologs), and (c) benign sequences that stress-test specificity (e.g., housekeeping genes shared between pathogenic and non-pathogenic organisms).

Performers: NIST, SBRC, computational biologists, immunologists, DNA providers, and tool developers.

Outputs: The benchmark against which all Phase II tools are evaluated.

**Item**	**Cost**	**Reference**
Personnel: 4 FTEs × 2 yr × $300K	$2.4M	2 bioinformaticians, 1 ML engineer (adversarial/paraphrased sequence generation), 1 biosecurity domain expert (National Institutes of Health, 2024; ZipRecruiter, 2024; National Institute of Allergy and Infectious Diseases, 2024; Congressional Research Service, 2024; Association of Public and Land-grant Universities, n.d.)
Sequence synthesis for test set	$0.5–1M	˜500–2,000 test sequences at varying lengths. At $0.10/bp average and 1–5 kb per construct: $50K–$1M depending on scale (Bio Basic, n.d.; Carlson, 2025; Twist Bioscience, 2025)
Data curation infrastructure	$0.3M	Database design, access control, versioning
**I.3 Subtotal**	**$3.2–3.7M**	

4.2 Phase II: Tools and Validation (Years 2–5, $31–40M)

4.2.1 Workstream II.1 — Computational tools

Activity: Developing or adapting screening algorithms against the Phase I definition. At least one team focused on adapting existing tools (FAST-NA, Common Mechanism, SecureDNA) to the new ontology; at least one developing PLM-based methods for detecting functional similarity despite sequence divergence; at least one exploring structure-prediction approaches. Tools should be stackable: fast methods (signature matching, exact-match lookup) screen everything; expensive methods (PLM embeddings, structure prediction) apply only to unresolved sequences.

Performers: 3–4 independent screening tool developer teams.

Outputs: One or more screening tools capable of detecting known threats and paraphrased threats (novel sequences with functions defined in the ontology).

**Item**	**Cost**	**Reference**
Personnel: 7 FTEs × 4 yr × $300K	$8.4M	3 ML/software engineers, 2 computational biologists, 1 senior biodefence AI/ML (BAIM) researcher, 1 DevOps/integration (National Institutes of Health, 2024; ZipRecruiter, 2024; National Institute of Allergy and Infectious Diseases, 2024; Congressional Research Service, 2024; Association of Public and Land-grant Universities, n.d.)
GPU compute for BAIM training & serving	$2–4M	Multiple training runs, hyperparameter sweeps, adversarial retraining. 2–4 major training campaigns per year at $200–500K each (GMI Cloud, 2025; RunPod, 2025). For reference, DeepSeek-V3 (671B params) trained for ~$6M (Intuition Labs, 2025); ADAPT models would be smaller but iterative
Software/API development & hardening	$0.5M	Screening tool APIs, integration testing, documentation
**II.1 Subtotal**	**$11–13M**	

4.2.2 Workstream II.2 — Experimental validation of AI-generated sequences

Activity: High-containment wet-lab work to synthesise and functionally characterise edge-case sequences: novel AI-generated sequences identified by II.3 red-teaming as potential evasions, and sequences where Phase II tools disagree and no ground truth exists.

Performers: Parts of this likely require a national laboratory or other high-containment facility.

Outputs: Annotation of ambiguous cases and feedback to tool developers (II.1) and the maintainers of the ontology (I.1).

Item	Cost	Reference
Personnel: 6 FTEs × 4 yr × $300K	$7.2M	2 synthetic biologists, 2 virologists/immunologists, 1 BSL-3 technician, 1 data analyst (National Institutes of Health, 2024; ZipRecruiter, 2024; National Institute of Allergy and Infectious Diseases, 2024; Congressional Research Service, 2024; Association of Public and Land-grant Universities, n.d.)
Gene synthesis of edge-case constructs	$1–3M	Hundreds of novel constructs per year. Standard synthesis at $0.10–0.20/bp; complex/non-standard at $0.25–0.60/bp (Bio Basic, n.d.; Carlson, 2025; Twist Bioscience, 2025). Rush orders and difficult sequences increase cost
Functional characterisation assays	$4–8M	Cell-based assays (infectivity, toxicity, host-range), potentially small animal models. Iterative: new constructs from II.3 feed continuously into II.2. CRO costs for BSL-3 work are premium (Target Discovery Institute, University of Oxford, n.d.; Bock et al., 2022)
BSL-3 facility access/maintenance	$1–2M	Operating costs ˜$90–129 per net square foot (nsf) (Tradeline, Inc., 2012, 2015). External facility access at ˜$200/day (Rush University Medical Center, n.d.); 5–7% annual inflation projected (Kansas State University Biosecurity Research Institute, n.d.). Budget assumes shared access across multiple partner institutions
**II.2 Subtotal**	**$13–20M**	

4.2.3 Workstream II.3 — Red-teaming and benchmarking

Activity: Independent evaluation of all tools against the Phase I test set, and adversarial testing with AI-designed sequences. Adversarial sequences of interest are passed to II.2 for experimental validation. Results feed back into tool refinement and ontology updates.

Performers: Computational biologists and biotech researchers not involved in earlier workstreams, with access to liaisons who have been.

Outputs: Benchmark results for all tools, candidate sequences for II.2 validation, recommendations for ontology revisions.

**Item**	**Cost**	**Reference**
Personnel: 5 FTEs × 4 yr × $300K	$6.0M	2 ML red-teamers, 1 computational biologist, 1 evaluation/benchmarking specialist, 1 biosecurity analyst (National Institutes of Health, 2024; ZipRecruiter, 2024; National Institute of Allergy and Infectious Diseases, 2024; Congressional Research Service, 2024; Association of Public and Land-grant Universities, n.d.)
Compute for adversarial sequence generation	$0.5–1M	Inference-heavy rather than training-heavy. Using LLMs and biological design tools to generate evasion sequences. At current inference prices ($0.27–$3/M tokens) (Intuition Labs, 2025)
**II.3 Subtotal**	**$6.5–7M**	

4.3 Continuous: Governance and Transition ($13.5–14M)

4.3.1 Workstream G.1 — Policy translation

Activity: Translating technical outputs into regulatory guidance for relevant bodies (in the U.S.: HHS, OSTP; in the UK: DSIT; internationally: IGSC, WHO). This includes mapping the ADAPT definition to existing frameworks (e.g., Executive Order requirements, IGSC protocols) and identifying gaps requiring new regulation.

Performers: A small standing biosecurity policy team, running throughout but intensifying in Years 4–5.

Outputs: Draft guidance documents, regulatory impact assessments, and briefing materials for policymakers.

Item	Cost	Reference
Personnel: 3 FTEs × 5 yr × $300K	$4.5M	2 policy researchers, 1 regulatory affairs/stakeholder liaison. Policy researchers may be at think tanks with lower overhead, making $300K conservative (National Institutes of Health, 2024; ZipRecruiter, 2024; National Institute of Allergy and Infectious Diseases, 2024; Congressional Research Service, 2024; Association of Public and Land-grant Universities, n.d.)
Stakeholder engagement & travel	$0.5M	Engagement with HHS, OSTP, DSIT, IGSC, WHO. Workshops, regulatory consultations, international convenings
**G.1 Subtotal**	**$5.0M**	

4.3.2 Workstream G.2 — Secure infrastructure

Activity: Design and deployment of hosting architecture for the ontology, test sets, and screening tools. The definition itself may pose an information hazard, so managed-access controls are required. For computationally expensive components (e.g., PLM-based classifiers), options include: federated deployment to provider sites with cryptographic verification, secure enclaves, or API-based access where providers submit sequence embeddings rather than raw sequences.

Performers: Security engineers, in coordination with tool developers and providers.

Outputs: Secure, privacy-preserving infrastructure enabling provider adoption without exposing threat-detection logic or proprietary sequences (Baum et al., 2026).

Item	Cost	Reference
Personnel: 4 FTEs × 5 yr × $300K	$6.0M	2 infosec/platform engineers, 1 backend engineer, 1 DevSecOps (National Institutes of Health, 2024; ZipRecruiter, 2024; National Institute of Allergy and Infectious Diseases, 2024; Congressional Research Service, 2024; Association of Public and Land-grant Universities, n.d.)
Secure cloud infrastructure	$2–2.5M	Controlled-access hosting; may require FedRAMP-equivalent or OFFICIAL-SENSITIVE (UK) hosting. HSMs, audit logging, access control. ˜$20–50K/mo over 5 years
Security audits & compliance	$0.5M	Penetration testing, compliance certification (SOC 2, ISO 27001, or equivalent)
**G.2 Subtotal**	**$8.5–9M**	

4.4 Programme management & coordination ($5M)

Item	Cost	Reference
Personnel: 3 FTEs × 5 yr × $300K	$4.5M	1 programme manager, 1 admin/finance, 1 comms/reporting (National Institutes of Health, 2024; ZipRecruiter, 2024; National Institute of Allergy and Infectious Diseases, 2024; Congressional Research Service, 2024; Association of Public and Land-grant Universities, n.d.)
Programme-level travel, annual reviews	$0.5M	PI meetings, annual programme reviews, reporting
**PM Subtotal**	**$5.0M**	

4.5 Total cost summary

Table S3. Total estimated cost of the ADAPT programme.

Category	Duration	Low	High
Phase I (I.1 + I.2 + I.3)	Years 1–2	$15M	$19M
Phase II (II.1 + II.2 + II.3)	Years 2–5	$31M	$40M
Governance (G.1 + G.2)	Years 1–5	$13.5M	$14M
Programme management	Years 1–5	$5M	$5M
**Total**	**5 years**	**$65M**	**$78M**

4.6 Programme comparators

DARPA NOW (Nucleic Acids On-Demand Worldwide): Up to $41M awarded to GE Research, Broad Institute, DNA Script and collaborators (Global Biodefense, 2021).

ARIA (UK): £800M over 5 years (2023–2028) (UK Parliament, 2022), with individual programmes at £20–57M each (UK Government, 2022). The Scaling Trust programme is £50M; the SRM/climate programme is £56.8M (ARIA, 2024).

DARPA: FY2024 enacted budget of $4.1B across ~100 programme managers (Defense Advanced Research Projects Agency, 2024). Average programme is $20–50M over its lifetime; flagship programmes can exceed $100M.

4.7 Cost assumptions

All costs are in 2025 USD. The GBP equivalent of $65–78M is approximately £50–60M at current exchange rates. Costs are benchmarked to U.S. institutional rates, which are higher than UK rates due to higher indirect cost recovery; this makes the estimate conservative for a UK-funded programme.

Fully loaded FTE cost (~$300K/yr): Assumes a blended base salary of ~$140K across postdocs ($65–80K) (National Institutes of Health, 2024; ZipRecruiter, 2024), mid-career researchers ($120–180K), senior PIs (up to NIH salary cap of ~$221K) (National Institute of Allergy and Infectious Diseases, 2024), and software engineers ($150–200K+). Benefits at ~30% of salary and indirect costs at ~50% of modified total direct costs (median U.S. university ICR of ~56%) (Congressional Research Service, 2024; Association of Public and Land-grant Universities, n.d.). Calculation: $140K $\times$ 1.30 $\times$ 1.50 $\approx$ $273K, rounded to $300K for inflation and geographic variation.

GPU compute: H100 GPUs at $2–8/hr depending on provider (2025 pricing) (GMI Cloud, 2025; RunPod, 2025). A single fine-tuning campaign on a 3B-parameter model costs ~$10–50K.

Gene synthesis: ~$0.09–0.20/bp for standard synthesis; $0.25–0.60/bp for complex sequences (Bio Basic, n.d.; Carlson, 2025; Twist Bioscience, 2025).

BSL-3 facility costs: Operating costs of ~$90–129/nsf (Tradeline, Inc., 2012, 2015). External facility access at ~$200/day (Rush University Medical Center, n.d.), with 5–7% annual inflation projected (Kansas

State University Biosecurity Research Institute, n.d.). These benchmarks are from 2012–2015 and likely understate current costs, but the budgeted range accounts for this.

REFERENCES

(2025). Defense advanced research projects agency: Fiscal year 2026 budget estimates https://comptroller.war.gov/Portals/45/Documents/defbudget/FY2026/budget_justification/pdfs/03_RDT_and_E/RDTE_Vol1_DARPA_MasterJustificationBook_PB_2026.pdf Accessed: 26 February 2026

[Dataset] ARIA (2024). Funding opportunities. https://www.aria.org.uk/funding-opportunities/. Accessed: 26 February 2026

[Dataset] Association of Public and Land-grant Universities (n.d.). Research facilities and administrative costs. https://www.aplu.org/our-work/4-policy-and-advocacy/research-and-science/research-facilities-administrative-costs/. Accessed: 26 February 2026

Balaji, A., Kille, B., Kappell, A. D., Godbold, G. D., Diep, M., Elworth, R. L., et al. (2022). Seqscreen: accurate and sensitive functional screening of pathogenic sequences via ensemble learning. *Genome biology* 23, 133

Battelle (2018). ThreatSEQ™ web service: Advanced DNA screening platform. *Product brief* https://www.crcfbt.org/docs/threatseq.pdf. Accessed: 25 February 2026

Baum, C., Berlips, J., Chen, W., Cozzarini, H., Cui, H., Damgård, I., et al. (2026). A system capable of verifiably and privately screening global dna synthesis. *National Science Review* , nwag103

[Dataset] Bio Basic (n.d.). Gene synthesis pricing. https://www.biobasic.com/genes-pricing/. Accessed: 26 February 2026

Bock, C., Datlinger, P., Chardon, F., Coelho, M. A., Dong, M. B., Lawson, K. A., et al. (2022). High-content CRISPR screening. *Nature Reviews Methods Primers* 2, 8. doi:10.1038/s43586-021-00093-4

California State Legislature (2021). Assembly Bill 70: Gene synthesis providers. *California State Legislature* https://leginfo.legislature.ca.gov/faces/billNavClient.xhtml?bill_id=202120220AB70. Accessed: 26 February 2026

California State Legislature (2022). Assembly Bill 1963: California State University and University of California: Gene synthesis providers. *California State Legislature* https://leginfo.legislature.ca.gov/faces/billNavClient.xhtml?bill_id=202120220AB1963. Accessed: 26 February 2026

[Dataset] Carlson, R. (2025). DNA synthesis and sequencing costs and productivity for 2025. http://www.synthesis.cc/synthesis/2025/5/dna-synthesis-and-sequencing-costs-and-productivity-for-2025. Accessed: 26 February 2026

[Dataset] Congressional Research Service (2024). Facilities and administrative costs in federal research grants. https://www.congress.gov/crs-product/R48540. Accessed: 26 February 2026

[Dataset] Defense Advanced Research Projects Agency (2024). About DARPA. https://www.darpa.mil/about. Accessed: 26 February 2026

DNA Script (2021). DNA Script partners with Moderna to develop on-demand vaccines and therapeutics for DARPA. *Press release* https://www.dnascript.com/press-releases/dna-script-partners-with-moderna-to-develop-on-demand-vaccines-and-therapeutics-for-darpa/. Accessed: 26 February 2026

Gemler, B. T., Mukherjee, C., Howland, C., Fullerton, P. A., Spurbeck, R. R., Catlin, L. A., et al. (2023). Ultraseq, a universal bioinformatic platform for information-based clinical metagenomics and beyond. *Microbiology Spectrum* 11, e04160–22

Global Biodefense (2021). DARPA awards nucleic acids on-demand (NOW) contract to GE Research and collaborators. *Global Biodefense* https://globalbiodefense.com/2021/03/02/darpa-awards-nucleic-acids-on-demand-now-contract-to-ge-research-and-collaborators/. Accessed: 26 February 2026

[Dataset] GMI Cloud (2025). How much do GPU cloud platforms cost for AI startups in 2025? https://www.gmicloud.ai/blog/how-much-do-gpu-cloud-platforms-cost-for-ai-startups-in-2025. Accessed: 26 February 2026

Godbold, G. D., Kappell, A. D., LeSassier, D. S., Treangen, T. J., and Ternus, K. L. (2022). Categorizing sequences of concern by function to better assess mechanisms of microbial pathogenesis. *Infection and immunity* 90, e00334–21

[Dataset] Intuition Labs (2025). DeepSeek inference cost explained. https://intuitionlabs.ai/articles/deepseek-inference-cost-explained. Accessed: 26 February 2026

[Dataset] Kansas State University Biosecurity Research Institute (n.d.). Schedule of charges. https://www.bri.k-state.edu/research/schedule-of-charges.html. Accessed: 26 February 2026

Maryland State Legislature (2021). House Bill 1256: Maryland Department of Health — gene synthesis providers and manufacturers of gene synthesis equipment — certification. *Maryland State Legislature* https://mgaleg.maryland.gov/mgawebsite/Legislation/Details/hb1256?ys=2021RS. Accessed: 26 February 2026

[Dataset] National Institute of Allergy and Infectious Diseases (2024). Salary cap and stipend levels. https://www.niaid.nih.gov/grants-contracts/salary-cap-stipends. Accessed: 26 February 2026

[Dataset] National Institutes of Health (2024). Ruth L. Kirschstein national research service award (NRSA) stipend levels, FY2024. https://www.nih.gov/news-events/news-releases/nih-increase-pay-levels-pre-postdoctoral-scholars-grantee-institutions. Accessed: 26 February 2026

National Science and Technology Council (2024). Framework for nucleic acid synthesis screening. *Office of Science and Technology Policy* https://bidenwhitehouse.archives.gov/ostp/news-updates/2024/04/29/framework-for-nucleic-acid-synthesis-screening/. Accessed: 26 February 2026

[Dataset] RunPod (2025). Top cloud GPU providers. https://www.runpod.io/articles/guides/top-cloud-gpu-providers. Accessed: 26 February 2026

[Dataset] Rush University Medical Center (n.d.). Rush BSL-3 biohazard containment core. https://www.rushu.rush.edu/research-rush-university/rush-core-laboratories/rush-bsl-3-biohazard-containment-core. Accessed: 26 February 2026

[Dataset] Target Discovery Institute, University of Oxford (n.d.). CRISPR pooled screening. https://www.tdi.ox.ac.uk/research/research/cellular-high-throughput-screening-hts/crispr-pooled-screening/crispr-loss-of-function-screening. Accessed: 26 February 2026

The White House (2023). Executive Order 14110: Safe, secure, and trustworthy development and use of artificial intelligence. *Federal Register* https://www.whitehouse.gov/briefing-room/presidential-actions/2023/10/30/executive-order-on-the-safe-secur

e-and-trustworthy-development-and-use-of-artificial-intelligence/. Accessed: 26 February 2026

The White House (2025). Executive order on improving the safety and security of biological research. *Federal Register* https://www.whitehouse.gov/presidential-actions/2025/05/improving-the-safety-and-security-of-biological-research/. Accessed: 26 February 2026

[Dataset] Tradeline, Inc. (2012). Benchmarking operational costs of containment facilities. https://www.tradelineinc.com/reports/2012-11/benchmarking-operational-costs-containment-facilities. Accessed: 26 February 2026

[Dataset] Tradeline, Inc. (2015). Operating cost benchmarks for biomedical research facilities. https://www.tradelineinc.com/reports/2015-2/operating-cost-benchmarks-biomedical-research-facilities. Accessed: 26 February 2026

Twist Bioscience (2019). Twist Bioscience adopts Battelle's ThreatSEQ™ DNA screening web service. *Press release*

[Dataset] Twist Bioscience (2025). Gene synthesis services. https://www.twistbioscience.com. Accessed: 26 February 2026

UK Government (2022). Advanced research and invention agency (ARIA): Statement of policy intent. *UK Government Policy Paper* https://www.gov.uk/government/publications/advanced-research-and-invention-agency-aria-statement-of-policy-intent/advanced-research-and-invention-agency-aria-policy-statement. Accessed: 26 February 2026

UK Parliament (2022). Advanced research and invention agency Act 2022. *UK Public General Acts* https://www.legislation.gov.uk/ukpga/2022/4/contents/enacted. Accessed: 26 February 2026

Wyschogrod, D., Manthey, J., Mitchell, T., Murphy, S., Clore, A., and Beal, J. (2022). Adapting malware detection to DNA screening. In *Proceedings of the 14th International Workshop on Bio-Design Automation (IWBDA)*

[Dataset] ZipRecruiter (2024). NIH postdoctoral salary survey. https://www.ziprecruiter.com/Salaries/Nih-Postdoctoral-Salary. Accessed: 26 February 2026
